# Finite Element Analysis of Manufacturing Deformation in Polymer Matrix Composites

**DOI:** 10.3390/ma17102228

**Published:** 2024-05-09

**Authors:** Thomas Singleton, Adil Saeed, Lloyd Strawbridge, Zulfiqar Ahmad Khan

**Affiliations:** 1 NanoCorr, Energy & Modelling (NCEM) Research Group, Bournemouth University, Poole BH12 5BB, UKzkhan@bournemouth.ac.uk (Z.A.K.); 2 Tods Technology, Wide Street, Portland DT5 2JP, UK; l.strawbridge@tods.co.uk

**Keywords:** elastic response, finite element analysis, composites, polymer, matrix, vacuum, pressure, stress, laminates

## Abstract

This paper introduces a unique finite element analysis (FEA) technique designed to predict elastic response in polymer matrix composites (PMCs). Extensive research has been conducted to model the manufacturing process of multiple ‘L’-shaped components, fabricated from SPRINT^TM^ materials (GLP 43 and GLP 96) at two thicknesses (15 mm and 25 mm). Three distinct FEA methodologies were utilised to determine the impact of thermal loads and rigid fixtures. An error deviation of 3.23% was recorded when comparing simulation results to experimental data, thereby validating the effectiveness of the FEA methodology.

## 1. Introduction

Polymer matrix composites (PMCs) are gaining significance in contemporary design, with global demand now surpassing 13 million tonnes annually [1]. This is due to their broad availability, high strength, low weight, and minimal maintenance properties, preferable within the aerospace, defence, and marine sectors [2,3,4].

Current manufacturing methods induce residual stresses within the laminate, leading to a form of product deformation known as elastic response [5,6,7]. This distortion has a significant impact on a company’s expenses as it necessitates product re-manufacturing, repairs, or disposal at rising landfill costs [8,9]. In 2018, the United Kingdom independently wasted 1200 tonnes and 6200 tonnes of carbon fibre-reinforced polymer (CFRP) and glass fibre-reinforced polymer (GFRP) respectively [9], with increased demand only escalating these quantities.

Unfortunately, disposal through landfills is currently unavoidable as the heterogeneous nature and matrix-fibre composition of PMCs hinder energy recovery strategies. This consequently fuels the global warming crisis and increases institutional carbon footprints, damaging a company’s market reputation and ability to adhere to international organisations such as ISO14000 [10] and UN sustainability goals.

This research aims to accelerate composite manufacturing and diminish industrial waste by creating a finite element analysis (FEA) technique that precisely forecasts elastic response in PMCs. By identifying deformation issues early in the design phase, engineers can adjust tooling specifications and construct products within the desired tolerances [11,12]. This approach helps reduce production waste and lower the costs associated with repairs, re-manufacturing, and disposal [7,8,9].

## 2. Initial Planning

**Figure 1:**
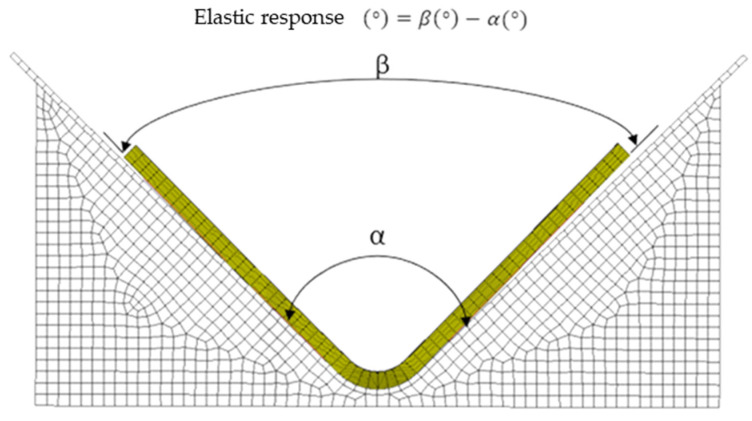
Measurement of elastic response.

This study will specifically evaluate two FEA methods that imitate the cure profile of multiple ‘L’-shaped components. Simulation one presents a simple, no-tooling approach [8], whilst simulation two introduces a more complex, tooling-included procedure [6,7]. All FEA results were compared with the experimental samples, while the elastic response was calculated as the difference between the interior tool angle α and the exterior component angle β, as shown in Figure 1. In this research, two SPRINT^TM^ materials GLP 43 and GLP 96 [13], with thicknesses of 15 mm and 25 mm (Table 1) were investigated for manufacturing distortion.

## 3. Background Research

### 3.1. Overview of Elastic Response

To produce consolidated components, vacuum bagging and autoclave methods subject were used to materials to a combination of high temperature, pressure, and chemical loads [5,12]. However, this process induces residual stresses within the laminate and initiates a form of deformation regarded as an elastic response [5,6,7,11,12]. For curved samples, the elastic response is defined as:

*“The difference between the corner angle of a specimen (under stress-free condition, after being extracted from its tool, post-cure at room temperature) and that of its tool (which represents the initial condition of the specimen before being extracted from its tool)”*

[12].

However, there is an exception; some authors suggest refining the use of this definition to only ‘L’-shaped components and propose using the term ‘warpage’ when referring to flat parts [8,11,14].

The main source of residual stress is the differing coefficients of thermal expansion (CTE) between the tooling and composite material [5,6,7,8,12,14,15], which causes the plies at the interface to expand during the initial stages of the cure cycle. Moreover, when the resin becomes viscous, it creates stress within the same plane that extends through the thickness of the laminate [6,8]. To amplify this idea, consider the thermal differences that arise between neighbouring plies, especially when using alternating orientations of anisotropic fibres [5,11]. 

Fibrous composites also sustain significant changes to their intrinsic characteristics during the curing process. The variance is most notable when the resin undergoes polymerisation, forming cross-links with neighbouring polymer chains. This transforms the molecular structure from an amorphous to a crystalline state and leads to a volume reduction in a phenomenon known as cure shrinkage [5,6,8,11,16,17]. When the vacuum pressure is finally released, the combined bending moments exert sufficient force to deform the component away from the tool [5,8].

### 3.2. Consequences of Elastic Response

Components that deviate from specified tolerance levels may lead to fitment issues during the assembly procedure. While some products might be forcibly connected, this can introduce internal stresses within the structure and ultimately decrease its lifespan [6,12]. Also, deformed parts must often be repaired or disposed of, both of which increase processing costs, delay delivery [7,8], and escalate a company’s carbon footprint,^14^,^15^].

### 3.3. Related Experiments

Past research has determined that manufacturing deformation is primarily influenced by the gradient of volumetric shrinkage within the laminate, caused by resin contracting more significantly near the tooling compared to the outer edges [14]. This demotes elastic response on thicker and larger samples as the cure shrinkage is distributed evenly in the plies [5,12,14,15]. However, it is worth noting that all previously published experiments employed different PMCs, rendering regression models and analytical calculations invalid as they are not comparable. Furthermore, these experiments did not account for the heterogeneous material selection, variation in ply orientations, or cure profile all of which, as mentioned earlier, regulate elastic response [11,15].

## 4. Methodology

### 4.1. Material Properties and Specimen Geometry

**Figure 2:**
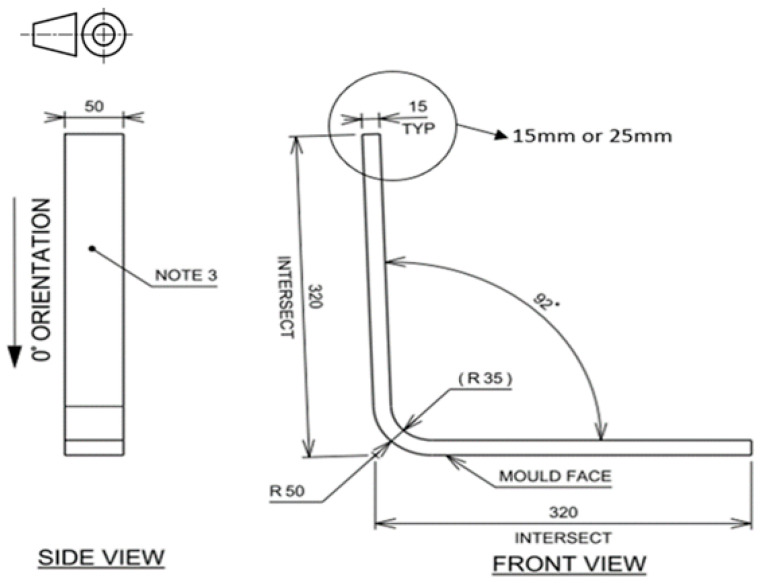
Profile of ‘L’ components (mm).

This research work utilised ‘L’-shaped components (Figure 2) manufactured from two SPRINT^TM^ materials, GLP 43 and GLP 96, at 15 mm and 25 mm thicknesses (Table 1 and Table 2). Both prepregs contained plies with quadriaxial stitching (0°, 90°, ±45°), enabling quasi-isotropic laminates to be produced without alternating individual orientations.

In this article, the mechanical properties of both materials are considered whilst thermal and resin characteristics were obtained from published data. The alternative solution of material testing was avoided due to higher costs and capacity restrictions.

### 4.2. Tangible Specimens

Since FEA results must be vindicated [6,7,12], tangible samples were manufactured through a vacuum bagging process with deformation being manually measured, as presented in Figure 1.

### 4.3. Layup and Processing

All samples were manufactured utilising the same concave aluminium tool, which was cleaned with acetone and coated with a release agent to eliminate residue and enhance extraction capabilities, before the lay-up process [15,19]. A surface film was then applied to achieve a smooth surface finish before adding layers of quadriaxial plies to create a quasi-isotropic laminate. The dimensions of each ply were 70 cm × 70 cm, with the quantity depending on the chosen material and sample thickness, as provided in Table 2.

All prepreg plies were aligned at a 0° orientation, with their length spanning the tool’s apex. Initially, the plies were oversized to compensate for both consolidation and resin leakage, but this was corrected by a post-cure trimming operation that redefined laminates to their final dimensions.

**Figure 3:**
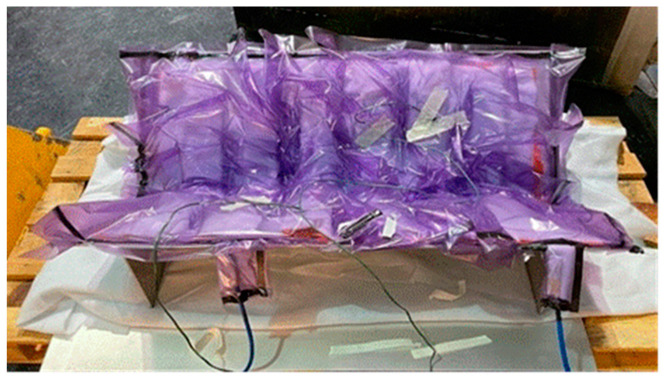
GFRP vacuum bagging.

A breather fabric was then used to cover the PMC components and a series of thermocouple sensors were set up to monitor the cure profile, specifically temperature and pressure fluctuations. Following this, a surface-bagging technique was employed on the tooling (Figure 3) and the entire assembly was placed inside a mechanical convection oven, a methodology utilised by [19].

### 4.4. Cure Profile

**Figure 4:**
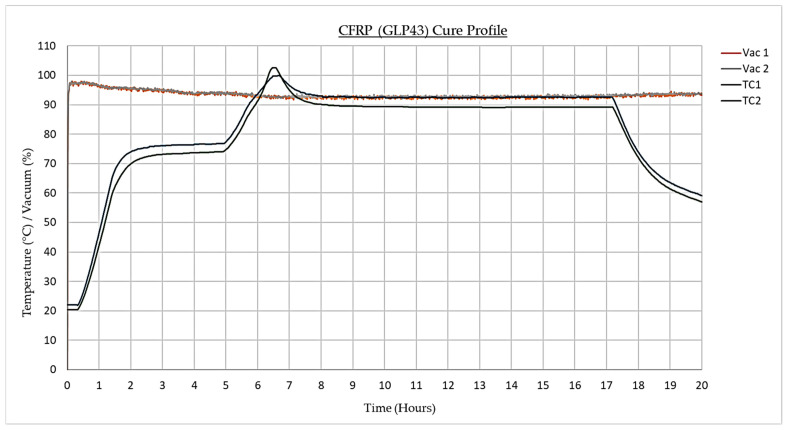
CFRP (GLP43) cure profile.

**Figure 5:**
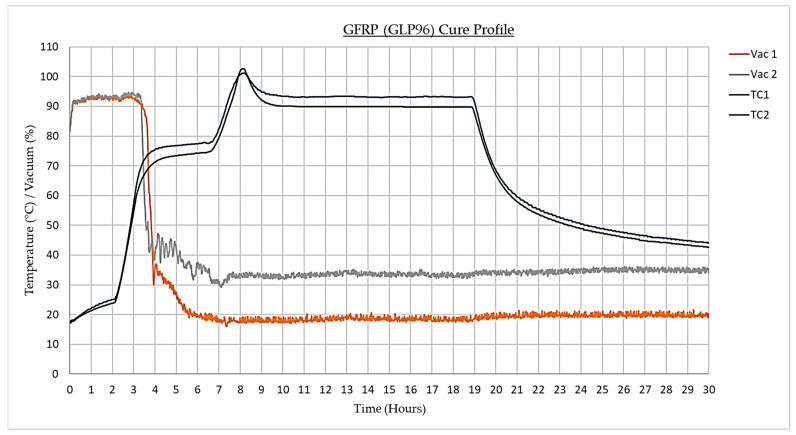
GFRP (GLP96) cure profile.

Both materials were exposed to identical cure cycles with dwell periods at 75 °C and 90 °C and a recommended pressure of 90,000 Pa (Figure 4). However, as presented in Figure 5, during the fabrication of the GFRP samples, the vacuum pressure decreased after 3 h. This was caused by the bag sealant melting, leading to the unwanted influx of air. To overcome this issue, an additional FEA simulation (simulation three) was developed to accurately model the realistic manufacturing process.

### 4.5. Measurement of Elastic Response Angle

The samples’ outer angles were measured twice using a digital protractor: first right after de-moulding, and then following the trimming process. The elastic response was measured by finding the variance between the interior tool angle (α) and the exterior component angle (β), as shown in Figure 1 and previously done by [5,8,11,12,14,15].

### 4.6. Quality Control and Consistency

To maintain uniformity, each component was produced in sets of three, as shown in Table 2. All samples possessed identical geometries and underwent the same fabrication processes, except for the manufacturing error previously mentioned. Additionally, curing GFRP and CFRP products concurrently ensured that any temperature or pressure fluctuations equally affected all three duplicates.

## 5. Finite Element Analysis (FEA)

### 5.1. Overview of FEA Procedure

FEA was conducted to perform the cure analysis by manipulating tabular data to suit a series of discrete models embedded within the software’s parameters; specifically, it exploits resin properties to quantify the kinetics of curing, shrinkage in volume, and laminate’s exothermic reaction. These resultant quantities then supplement conventional equations for heat transfer and stress deformation [16].

In other words, PMC fabrication was simulated by initiating a cure thermal mechanically coupled analysis [16] which augments a conventional heat transfer analysis by incorporating the additional influence of composite curing.

### 5.2. Discretisation

**Figure 6:**
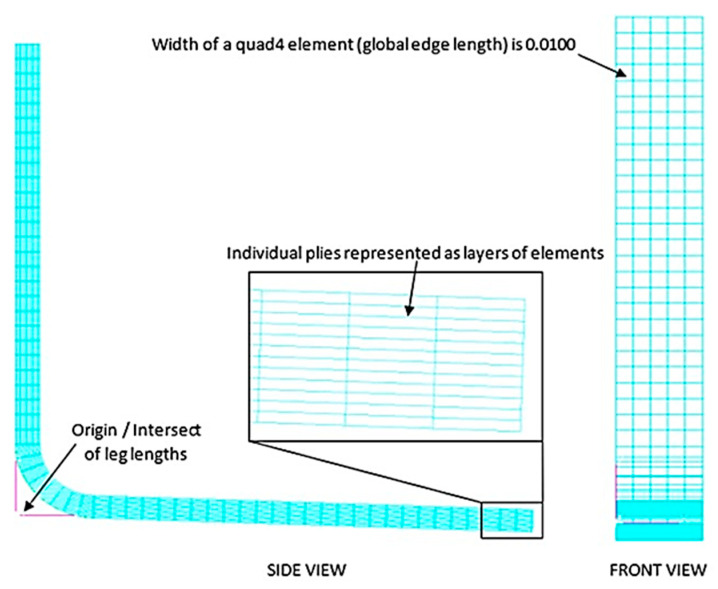
Optimum mesh on part 8010-1K.

**Figure 7:**
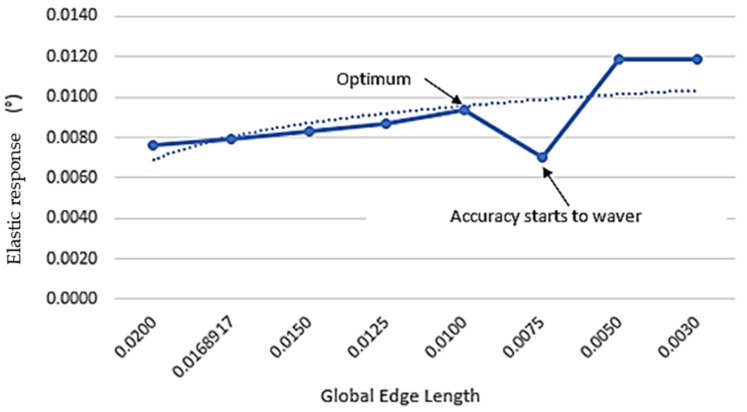
Elastic response in relation to global edge length.

**Figure 8:**
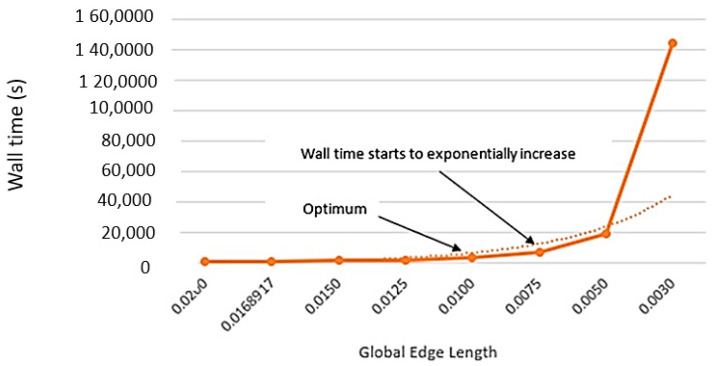
Wall time in relation to global edge length.

In the pre-processing stage, CAD models were converted into shell elements. The outer surface of the ‘L’ components was meshed into a series of quad-4 elements and subsequently extruded across the laminate thickness whilst segmenting individual layers [20] shown in (Figure 6). Duplicate nodes were then removed, and element coordinate systems were adjusted to align with the component contours. By completing a mesh convergence analysis, an optimal global edge length (0.0100) could be achieved [21] as shown in Figure 7 and Figure 8.

### 5.3. FEA Simulation One Methodology

Simulation one replicates the cure profile without processing the metallic tooling [8]. The procedure adopts two thermal–structural load cases: the first represents the initial 1755 min, while the second imitates the vacuum release and the final 3 min.

**Figure 9:**
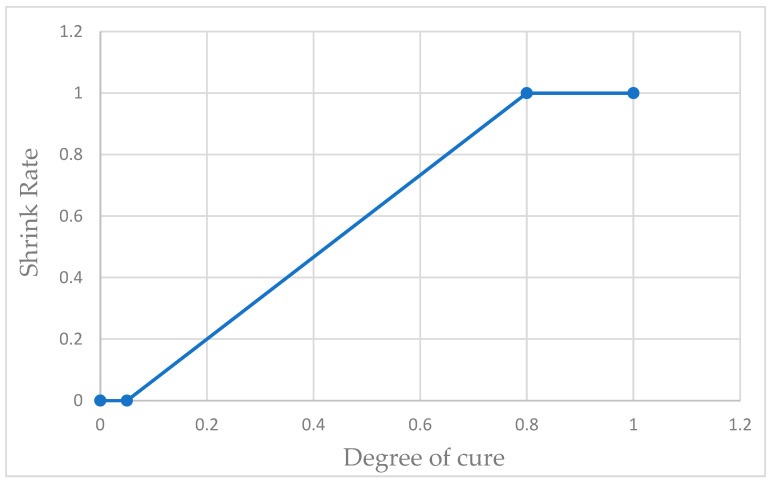
Polymerisation in relation to cure degree.

**Figure 10:**
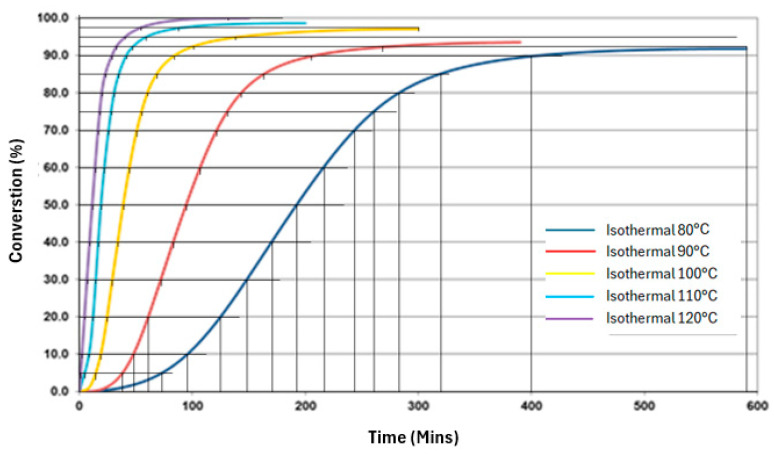
Rate of cure in relation to temperature, adopted from [22].

To begin, the material properties were entered into an analytical tool; shrinkage in volume, exothermic reaction, and rate of cure were controlled graphically, as shown in Figure 9 and Figure 10 [17,22]. The internal program automatically translates this tabular data into workable equations.

**Figure 11:**
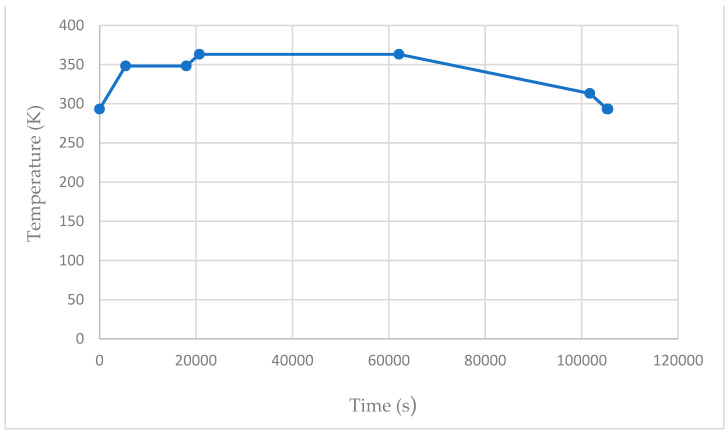
Oven temperature in relation to time.

Next, a face load of 90,000 Pa was applied to all exterior elements and assigned to load case 1 (replicating the vacuum pressure) whilst a time-dependent temperature (Figure 11) was enacted upon all nodes and allocated to load cases 1 and 2. These temperature measurements reflect the radiation emitted by the cavity and simulate the ideal manufacturing conditions (Figure 4). The emissivity for both materials was allocated a value of 1 to guarantee effective heat absorption.

**Figure 12:**
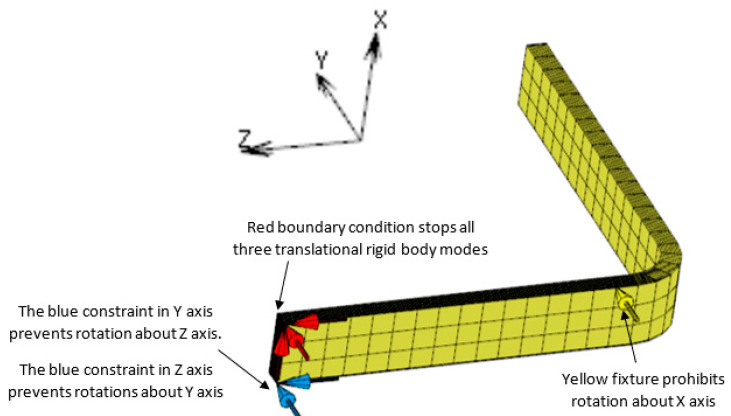
Load case 2 constraints [23].

Penultimately, components were constrained using nodal fixtures [23]. In load case 1, to restrict displacement, the exterior nodes were rigidly constrained in all directions. In load case 2, constraints were applied to restrict translation but allow thermal expansion and the generation of bending moments (Figure 12). An initial degree of cure (0% a minute) and starting oven temperature (20 °C) were then applied to define the first time-step.

### 5.4. FEA Simulation Two Methodology

**Figure 13:**
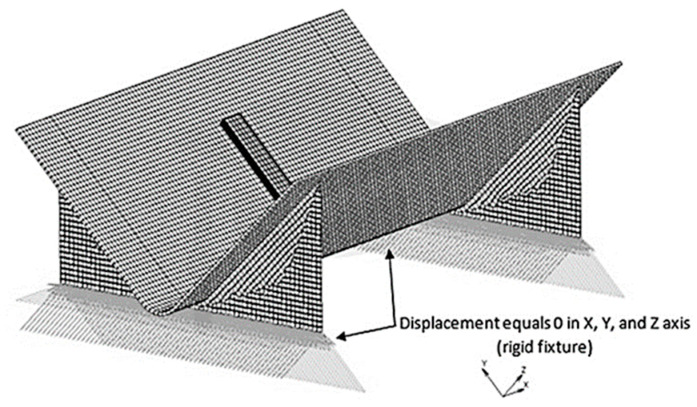
Aluminium Tooling Rigid Constraints.

Simulation two improves the FEA analysis by incorporating a tool part interface, also reported by other studies [6,7]. The discretised ‘L’ components were positioned to lay upon the metallic mould, as shown in Figure 13 before the aluminium tooling was rigidly fixed at multiple nodes to prevent any translational or rotational effects. After locating the models, two contact bodies were created. In both interactions, the contact conditions were defined as ‘touching’ with an exceptionally high heat transfer coefficient. However, in load case 1, the interaction used default settings, while in load case 2 the interaction was reconfigured to incorporate an extremely low separation force (0.0001 N). These parameters were chosen to facilitate heat conduction through the interface and to ensure that the composite components only distorted when the bending moments exceeded opposing forces, specifically those arising from the vacuum. A gravitational force was also applied to replicate real-world scenarios and to prevent unrealistic movement when releasing the vacuum pressure.

### 5.5. FEA Simulation Three Methodology

**Figure 14:**
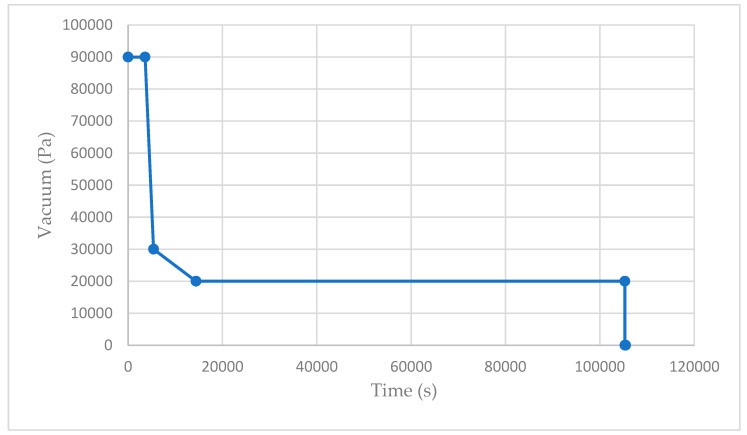
Vacuum pressure (failed) in relation to time.

Since the GFRP samples were manufactured with a diminishing vacuum (Figure 5), the initial FEA results did not align with the experimental data. As a result, a third simulation was developed to accurately mimic the real-life fabrication. While all other boundary conditions remained consistent with simulation two, there was a specific alteration made to the vacuum pressure. In this case, the vacuum pressure was modified to decrease from 90,000 Pa to 20,000 Pa over a period of three hours, as presented in Figure 14.

### 5.6. Digital Elastic Response Measurement

The elastic response was measured as the difference between α (internal tool) and β (external component) angles Figure 1 [5,8,11,12,14,15] shown in Figure 1 to find the digital product’s external angle, a curve was created on the outer surface of the ‘L’ component. These curves were then intersected and the resulting angle was calculated.

**Figure 15:**
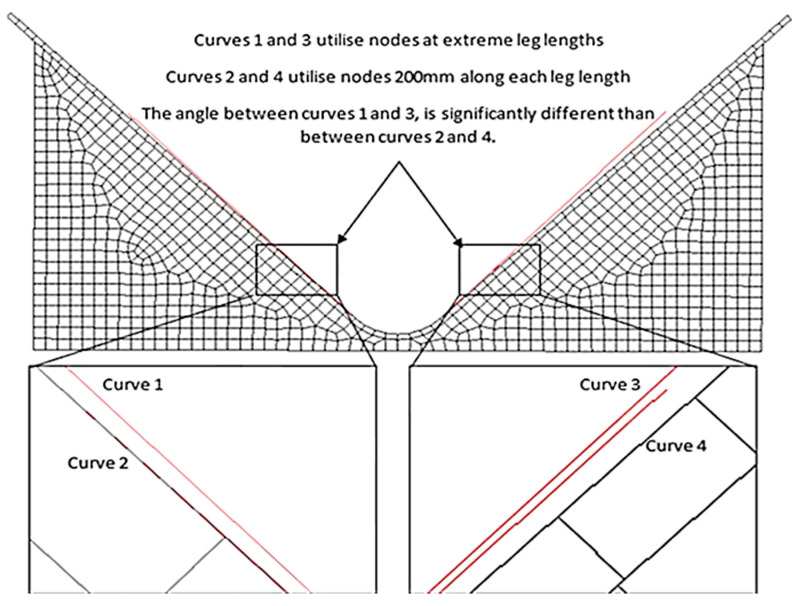
The consequence of different curve lengths.

Unfortunately, as the curve length increased, the exterior angle and subsequent elastic response decreased (despite the time step and geometry remaining constant). This was due to the leg lengths of the composite product deforming more at their extremities. Subsequently, curves using these nodal positions had different vectors, highlighted in Figure 15.

**Figure 16:**
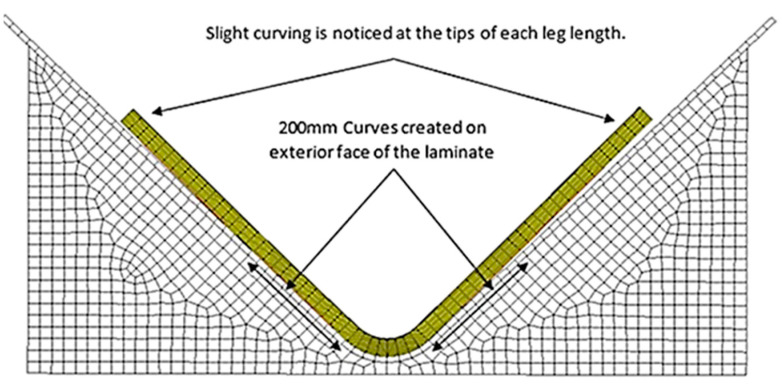
The final result of Simulation Two, Part 8010-1K.

To overcome this issue, it was decided that 200 mm curves should be created, starting from the intersection, to match the lengths of the digital protractor; this way, both physical and digital results would employ corresponding datums (Figure 16).

## 6. Results

### 6.1. Experimental Results

As previously discussed, the physical samples underwent measurement twice, first after the de-moulding process and then following the trimming operation. The average angle and subsequent elastic response were computed utilising Equations (1) and (2), respectively [12]. Given the known interior tooling angle (α) of 92°, Equation (2) is further refined into Equation (3). The results are provided in Table 3 and Table 4 below.

(1) $$Elastic\;Response\left({}^{\circ}\right)=\frac{\Sigma angles\;from\;all\;samples\left({}^{\circ}\right)}{Number\;of\;samples}$$

(2) $$Elastic\;Response\left({}^{\circ}\right)=\beta \left({}^{\circ}\right)-\alpha ({}^{\circ})$$

(3) $$Springback\left({}^{\circ}\right)=Exterior\;componant\;angle\left({}^{\circ}\right)-92{}^{\circ}$$

### 6.2. Simulation Results

Like the experimental samples, the FEA results calculated elastic response (at the final step-time) using Equation (3), before storing results in Table 5 and Table 6. To reiterate, simulation three imitates the vacuum pressure alleviating during GFRP manufacture (part numbers 8010-1K, 8010-2K). A concise comparison of the experimental and simulation results is provided in Table 7.

### 6.3. Error Deviation of Results

To evaluate the accuracy of the FEA simulations, the digital results were compared directly to the post-trim angles ascertained from the experimental data [6]. Equation (4) was then used to determine the error deviation (ED) between the two, with the results concluded in Table 8. The discrepancy of 108.83 in simulation one occurred because the simulation and physical specimens moved in opposite directions.

(4) $$Error\;deviation\left(\%\right)=[\frac{Tangible\;results\left({}^{\circ}\right)-Simulation\;results\left({}^{\circ}\right)}{Tangible\;Results\left({}^{\circ}\right)}]\times 100$$

## 7. Discussion

### 7.1. Accuracy of Results

#### 7.1.1. Simulation One

Simulation one was inadequate in forecasting the deformation caused by the vacuum bagging process since no distortion was detected (Table 5), resulting in the absence of recorded elastic response (Table 6 and Table 7). These results were a consequence of inaccurate guidance from the simulation software regarding applying minimal fixtures for securing the component ‘L’ [23] as shown in (Figure 12). However, Kurowski [24] opposes this as follows:

*“If a structure is not fully supported, it can move as a rigid body without any deformation. A structure with no support has six rigid body modes”*

[24].

Table 6 and Table 7 show minor distortions for products 8010-3K and 8010-4K. However, this is explained by a combination of discretisation, idealisation, and calculation errors, rather than an accurate representation of the working methodology [24]. Table 8 reinforces this theory by evidencing substantial error discrepancies, specifically with component 8010-3K exhibiting deformations in the opposite direction compared to the experimental samples.

#### 7.1.2. Simulation Two 

Simulation two accurately predicted elastic response to the nearest degree (Table 7). However, the results reveal limitations of the methodology, as some findings portray relatively small elastic response values compared to their experimental counterparts. This is not the case for component 8010-1K, where the simulation was conservative, showing notable elastic response (Table 7).

This method also led to error deviations, as indicated in Table 8. The inaccuracies for the GFRP components (parts 8010-1K, 8010-2K) can be attributed to the FEA applying a complete vacuum, unlike the experimental technique. In contrast, CFRP components (8010-3K and 8010-4K) used incorrect material characteristics sourced from the published data. This highlights some restrictions of the methodology as material properties have a substantial impact on deformation [5,6,7,8,12,14,15].

#### 7.1.3. Simulation Three 

Through the integration of the experimental procedures, simulation three enhanced the FEA process by aligning the elastic response results more closely with those of the actual specimens. Notably, components 8010-2K exhibited a nominal error deviation of 3.23% (Table 8). It is important to highlight that the accuracy of configuration 8010-1K decreased when realistic parameters were incorporated (Table 8).

### 7.2. Influence of Material Selection on Elastic Response

The simulation results lack accuracy in assessing the influence of material choice on elastic response because of its dependability on imprecise material properties sourced from published research. Nevertheless, the experimental findings (Table 7) suggest that CFRP (GLP 43) is more susceptible to manufacturing deformation than GFRP (GLP 96). This susceptibility can be attributed to the larger coefficient of thermal expansion disparity between CFRP and aluminium tooling [6,8].

### 7.3. Laminate Thickness’ Influence on Elastic Response

The reduction in elastic response is inversely proportional to the laminate thickness due to the even distribution of cure shrinkage that occurs across all plies [5,12,14]. However, this project provides uncertain findings regarding any correlation. The experimental data (Table 7) for CFRP components (8010-3K, 8010-4K) align with the established literature, while GFRP samples (8010-1K, 8010-2K) suggest that elastic response increases with thickness.

This contrast is attributed to budget limitations for sample sizes, which, as indicated by [25], heightened the risk of type 2 errors and led to the unjustifiable hypothesis rejection. To address this issue and obtain a more accurate average, it is advisable to increase the number of physical specimens [25].

### 7.4. Further Evaluation

#### 7.4.1. The Impact of Trimming 

The trimming process alleviates residual stress stored within the laminate (Table 7). Consequently, by releasing bending moments the component is commonly distorted and elastic response is exacerbated as observed by most of the test samples (Table 3); this is a key finding as the process is standard practice within PMC manufacturing.

#### 7.4.2. The Impact of Vacuum Pressure

When analysing simulations two and three involving GFRP components (specifically, specimens 8010-1K and 8010-2K, as shown in Table 6), it is apparent that maintaining a consistent vacuum has a notable effect on reducing elastic response. This effect is attributed to the pressure applied during resin polymerisation, which consolidates the laminate and persists until all residual stress is solidified within the crystalline structure. Subsequently, the component remains less susceptible to distortion from external forces, with significant bending moments being the primary factor affecting its shape. Conversely, when the vacuum is intermittently interrupted, the pressure on the reinforcement decreases significantly (to 20,000 Pa), allowing weaker forces to deform the part during the curing process and thus amplifying the elastic response effect [5,8].

#### 7.4.3. Identification of Stress

**Figure 17:**
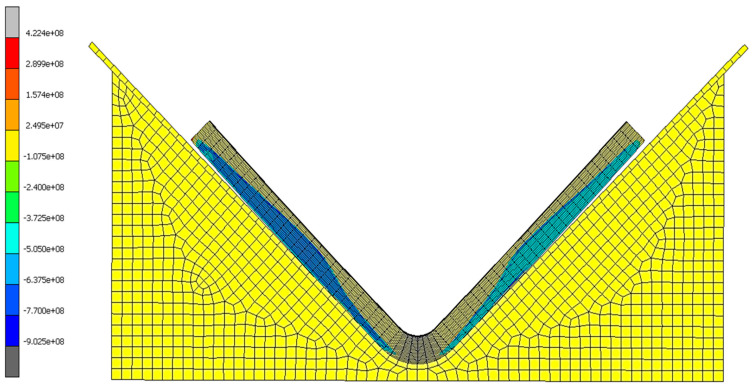
Simulation three results of normal stress at the final step time for 8010-2K.

**Figure 18:**
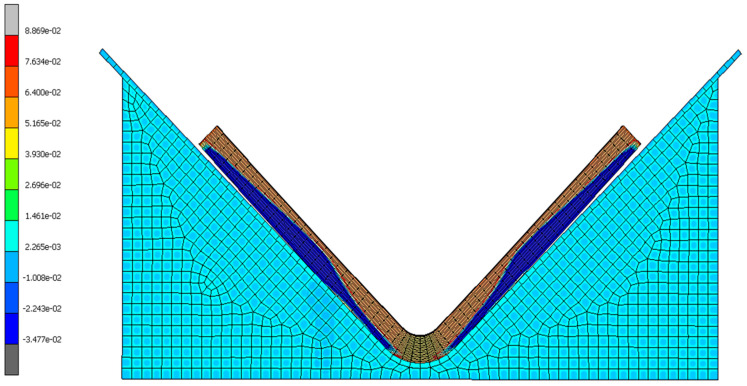
Simulation three results of volumetric cure shrinkage at the final step time for 8010-2K.

**Figure 19:**
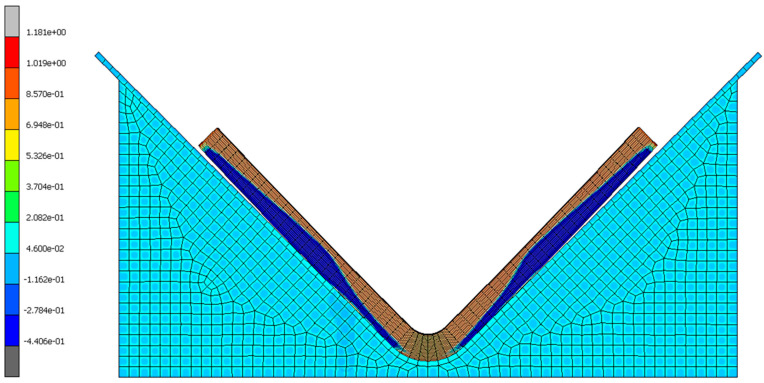
Simulation three results of cure degree at the final step time for 8010-2K.

By evaluating this project’s most successful simulation (part 8010-2K, simulation three), the origins of elastic response were determined. The normal stress and volumetric cure shrinkage infer a significant correlation (Figure 17 and Figure 18), verifying that the residual stress is due to the phase change from an amorphous to a crystalline molecular structure [5,6,8,11,16,17]. Figure 18 and Figure 19 demonstrate that the interface between the tooling and composite hinders the cure rate of the adjacent resin due to the heat-conducting properties of the metallic material leading to a reduction in kinetic energy. The finite element analysis results also indicate that plies near the tool show a negative shrink in volume (Figure 18), causing a negative state of stress (Figure 17). This occurrence is explained within other studies [6,8] where authors mention that plies located at the interface expand during the process of curing due to the viscous state of resin. This expansion generates an in-plane stress that extends across the entire thickness of the laminate [6,8].

## 8. Conclusions

By evaluating three semi-empirical simulations, a new methodology has been developed for predicting elastic response in PMCs, specifically when using relevant FEA software.

It was confirmed that the most effective approach for constraining CAD models involved implementing a tool-part interface. This method prevented translation in all six rigid body modes [24] and also incorporated heat transfer through conduction, mirroring real-world manufacturing processes. The importance of replicating physical manufacturing was underscored by conducting tests with a range of vacuum pressures, with simulation three achieving an impressive minimal error deviation of 3.23%.

Furthermore, by validating experimental data with finite element analysis (FEA), this research enhances current information regarding the polymer matrix composite (PMC), elastic response, and the following variables:

Type of material: The larger the CTE disparity between the laminate and tooling the more subjective composite products are to manufacturing deformation [6,8]. Thus, CFRP responses/springs back more than CFRP.

Trimming operation: the post-cure trimming of composites helps to relieve residual stresses stored within the laminate, leading to the generation of bending moments that induce deformation.

Vacuum pressure: maintaining a high vacuum pressure during fabrication constrains products during resin polymerisation and reduces the influence of cure shrinkage.

Causes of elastic response: the attained results strongly conclude there is a significant connection between volumetric cure shrinkage and stress concentration, supporting the verdict of supplementary articles [5,6,8,11].

## Figures and Tables

**Table 1:** Overview of the initial plan.

Component Identity	Fibre	Matrix	GLP	Thickness (mm)
FEA Simulation 1: No Tooling				
8010-1K	QE-1174	WT-93	96	15
8010-2K	QE-1174	WT-93	96	25
8010-3K	QC-800	ST-94	43	15
8010-4K	QC-800	ST-94	43	25
FEA Simulation 2: Inclusive of Tooling				
8010-1K	QE-1174	WT-93	96	15
8010-2K	QE-1174	WT-93	96	25
8010-3K	QC-800	ST-94	43	15
8010-4K	QC-800	ST-94	43	25
Definition of Fibre Matrix by Gurit [13]				
QE-1174	Quadriaxial Glass weight 1174 g/m^2^			
QC-800	Quadriaxial Carbon weight 800 g/m^2^			
WT-93	Resin Grade			
ST-94	Resin Grade			
Material’s Code				
GLP 96	QE-1174 and WT93 combination			
GLP 43	QC-800 and ST94 combination			

**Table 2:** Ply layup of all components.

Component Identity	GLP	Thickness (mm)	No. of Plies	Ply Orientation	No. of Specimen
8010-1K	96	15	12	0°	3
8010-2K	96	25	21	0°	3
8010-3K	43	15	18	0°	3
8010-4K	43	25	30	0°	3

**Table 3:** Measured angles from tangible specimens.

Component Identity	Specimen	Pre-Trim Angle (°)	Pre-Trim Mean (°)	Post-Trim Angle (°)	Post-Trim Mean (°)
8010-1K	1	91.2	91.27	91.1	91.13
	2	91.3		91.2	
	3	91.3		91.1	
8010-2K	1	91.1	91.13	91.0	91.07
	2	91.0		90.9	
	3	91.3		91.3	
8010-3K	1	90.8	90.83	90.7	90.80
	2	90.9		90.9	
	3	90.8		90.8	
8010-4K	1	90.9	91.07	90.9	91.00
	2	91.3		91.2	
	3	91.0		90.9	

**Table 4:** Calculated Elastic Response from tangible samples.

Component Identity	Pre-Trim Mean Angle (°)	Pre-Trim Mean Elastic Response (°)	Post-Trim Mean Angle (°)	Post-Trim Mean Elastic Response (°)
8010-1K	91.27	−0.73	91.13	−0.87
8010-2K	91.13	−0.87	91.07	−0.93
8010-3K	90.83	−1.17	90.80	−1.20
8010-4K	91.07	−0.93	91.00	−1.00

**Table 5:** Measured angles from simulations.

Component Identity	Simulation One Final Angle (°)	Simulation Two Final Angle (°)	Simulation Three Final Angle (°)
8010-1K	92.00	90.93	90.70
8010-2K	92.00	91.15	91.10
8010-3K	92.01	91.48	-
8010-4K	91.99	91.23	-

**Table 6:** Calculated elastic response from simulations.

Component Identity	Simulation One Final Elastic Response (°)	Simulation Two Final Elastic Response (°)	Simulation Three Final Elastic Response (°)
8010-1K	0.00	−1.07	−1.30
8010-2K	0.00	−0.85	−0.90
8010-3K	0.01	−0.52	-
8010-4K	−0.01	−0.77	-

**Table 7:** Comparison of tangible and simulated Elastic Response.

Component Identity	Pre-Trim (°)	Post-Trim (°)	Simulation One (°)	Simulation Two (°)	Simulation Three (°)
8010-1K	−0.73	−0.87	0.00	−1.07	−1.30
8010-2K	−0.87	−0.93	0.00	−0.85	−0.90
8010-3K	−1.17	−1.20	0.01	−0.52	-
8010-4K	−0.93	−1.00	−0.01	−0.77	-

**Table 8:** Error deviation (ED) of simulations.

Component Identity	Simulation One ED (%)	Simulation Two ED (%)	Simulation Three ED (%)
8010-1K	100.00	−22.99	−49.43
8010-2K	100.00	8.60	3.23
8010-3K	108.83	56.67	-
8010-4K	99.00	23.00	-

## Data Availability

Data are contained within the article.
